# Expressing *OsMPK4* Impairs Plant Growth but Enhances the Resistance of Rice to the Striped Stem Borer *Chilo suppressalis*

**DOI:** 10.3390/ijms19041182

**Published:** 2018-04-13

**Authors:** Xiaoli Liu, Jiancai Li, Liping Xu, Qi Wang, Yonggen Lou

**Affiliations:** State Key Laboratory of Rice Biology & Ministry of Agriculture Key Lab of Agricultural Entomology, Institute of Insect Sciences, Zhejiang University, Hangzhou 310058, China; 21216107@zju.edu.cn (X.L.); jiancai.2007@163.com (J.L.); 21616161@zju.edu.cn (L.X.); q.wang302@gmail.com (Q.W.)

**Keywords:** rice, *Chilo suppressalis*, mitogen-activated protein kinase 4, jasmonic acid, salicylic acid, ethylene, herbivore-induced defense response

## Abstract

Mitogen-activated protein kinases (MPKs) play a central role not only in plant growth and development, but also in plant responses to abiotic and biotic stresses, including pathogens. Yet, their role in herbivore-induced plant defenses and their underlying mechanisms remain largely unknown. Here, we cloned a rice MPK gene, *OsMPK4*, whose expression was induced by mechanical wounding, infestation of the striped stem borer (SSB) *Chilo suppressalis*, and treatment with jasmonic acid (JA), but not by treatment with salicylic acid (SA). The overexpression of *OsMPK4* (oe-MPK4) enhanced constitutive and/or SSB-induced levels of JA, jasmonoyl-l-isoleucine (JA-Ile), ethylene (ET), and SA, as well as the activity of elicited trypsin proteinase inhibitors (TrypPIs), and reduced SSB performance. On the other hand, compared to wild-type plants, oe-MPK4 lines in the greenhouse showed growth retardation. These findings suggest that *OsMPK4*, by regulating JA-, ET-, and SA-mediated signaling pathways, functions as a positive regulator of rice resistance to the SSB and a negative regulator of rice growth.

## 1. Introduction

In their natural habitats, plants often face infestations by various herbivores. To protect themselves, plants have developed constitutive and induced defensive mechanisms [1,2,3]. Induced defenses are made when a plant recognizes damage-related signals from herbivores. In response to the signals, plants activate a defense-related signaling network consisting mainly of jasmonic acid (JA)-, salicylic acid (SA)-, and ethylene (ET)-mediated pathways, and induce the production of defensive compounds; as a result, herbivore resistance in plants increases [4,5,6,7]. During this process, mitogen-activated protein kinase (MPK) cascades play vital roles in the signaling network, functioning upstream and downstream of defense-related signaling pathways [8,9,10,11].

A typical MPK cascade comprises three modules: MPKs, MPK kinases (MPKKs or MEKs), and MPKK kinases (MPKKKs or MEKKs), all of which are evolutionarily conserved in all eukaryotes [12]. These modules are sequentially activated by the dual phosphorylation of threonine and tyrosine residues in a TxY (x = D or E) motif located in their kinase catalytic activation loop. By activating the module MPKs, which subsequently phosphorylate some transcription factors and enzymes, and thereby trigger downstream signaling components or pathways, MPK cascades have been reported to play central roles in plant growth, development, and responses to abiotic and biotic stresses, including defense responses to pathogens and herbivores [8,13,14,15,16,17]. In *Arabidopsis thaliana*, for instance, MPK3 and MPK6 directly enhance the activity of 1-amino-cyclopropane-1-carboxylic acid synthase 6 (ACS6) and ACS2 at both transcriptional and protein levels, and thereby lead to the increase in the production of ET [18,19]. In *Nicotiana attenuata*, the wound-induced protein kinase (WIPK) and salicylic acid-induced protein kinase (SIPK) (orthologs of *Arabidopsis thaliana* MPK3 (AtMPK3) and AtMPK6, respectively) regulate JA- and SA-mediated signaling pathways, as well as herbivore-induced defense responses [9]. In rice, silencing *OsMPK3* (an ortholog of *AtMPK3*, also known as *OsBIMK1*) reduces the level of herbivore-elicited JA and the activity of trypsin protease inhibitors (TrypPIs), which enhances its resistance to the striped stem borer (SSB) *Chilo suppressalis* [10]. Moreover, OsMPK3 has also been found to negatively regulate the resistance of rice to *Magnaporthe oryzae* and positively regulate the plant’s tolerance to both drought and submergence [20,21].

MPK4, which acts downstream of the MEKK1–MEK1/MEK2 cascade, is one of the best-characterized MPKs. In Arabidopsis, MPK4 exists in the nucleus in a ternary complex with MAP KINASE 4 SUBSTRATE1 (MKS1) and the WRKY transcription factor WRKY33; these inhibit the extent to which WRKY33 can act as a transcription factor. Upon stimulation, MPK4 is activated and MKS1 is phosphorylated, and, subsequently, both MKS1 and WRKY33 are released from MPK4; the released WRKY33 then regulates the expression of target genes [22,23]. MPK4 is believed to act as a negative regulator of plant immunity to pathogens and a positive regulator of plant growth [24,25,26,27]. In Arabidopsis, for example, the *mpk4* mutant is severely dwarfed, and exhibits elevated SA levels and enhanced resistance to biotrophic pathogens [28,29,30]. Similarly, in soybean (*Glycine max*), silencing *GmMPK4* enhances SA and H_2_O_2_ accumulation, and plant resistance to downy mildew and to the soybean mosaic virus, but reduces plant growth [26]. By contrast, in rice, OsMPK4 has been reported to act both as an activator and a repressor of plant resistance to *Xanthomonas oryzae* pv *oryzae* (*Xoo*) [31]. Recently, a few studies have revealed that MPK4 also plays an important role in herbivore-induced defense responses. In *N. attenuata*, silencing *MPK4* elevates elicited levels of JA and jasmonoyl-l-isoleucine (JA-Ile); silencing *MPK4* also activates a JA-independent defense pathway, which in turn increases the resistance of plants to *Manduca sexta* [32]. In summary, these new findings shed light on the role of MPK4 in herbivore-induced plant defenses, a role which has until recently remained largely unexplored.

Rice, a staple food worldwide, is attacked by many insect pests [33], among which SSB is one of the most serious. Previous studies in rice have revealed that SSB infestation induces the biosynthesis of JA, JA-Ile, SA, and ET; these compounds subsequently modulate defense responses, including the production of herbivore-induced volatiles and an increase in the activity of TrypPIs [34,35,36,37,38]. Given the key role of MPK4 in plant defenses, we isolated the rice MPK4 gene, *OsMPK4* (TIGR ID: *Os10g38950*, the homologue of Arabidopsis *MPK4* and tobacco *MPK4*; also known as *OsMPK6*), which belongs to subgroup B of the MPK family and harbors a well-conserved TEY motif, as well as the evolutionarily conserved C-terminal common docking domain [39], and we characterized its role in herbivore-induced defense responses. By combining molecular biology, inverse genetics, chemical analysis, and bioassays, we show that *OsMPK4* is induced by mechanical wounding and herbivore attack. The overexpression of *OsMPK4* increases basal and/or SSB-induced levels of JA, JA-Ile, ET, and SA, as well as the activity of TrypPIs; in response, the performance of SSB larvae is reduced. Moreover, lines overexpressing *OsMPK4* exhibit reduced size. All these findings suggest that OsMPK4 acts as a positive modulator of herbivore-elicited defense responses and a negative mediator of plant growth in rice.

## 2. Results

### 2.1. Mechanical Wounding, Striped Stem Borer (SSB) Infestation, and Jasmonic Acid (JA) Treatment Induce Expression of OsMPK4

We screened rice plants for herbivore-induced transcripts using rice microarrays and found that one MPK gene, *OsMPK4*, was upregulated after SSB infestation [40]. The full-length cDNA of the *OsMPK4*, including an open reading frame (ORF) of 1131 bp, was obtained by reverse transcription polymerase chain reaction (PCR) (Figure S1). Phylogenetic analysis of some MPKs in subgroup B from different species revealed that OsMPK4 is homologous to BdMPK6 in *Brachypodium distachyon*, to SiMPK6 in *Setaria italica* and to SbMPK6 in *Sorghum bicolor*; all of these proteins share more than 94% amino acid sequence identity with OsMPK4. OsMPK4 also shows homologous to well characterized MPK4 in *Arabidopsis thaliana* (AtMPK4) [22], *Brassica napus* (BnMPK4) [41], and *Nicotiana tabacum* (NtMPK4) [42] (Figure S2), whose amino acid sequence similarities to OsMPK4 were 81.12%, 82.18%, and 81.91%, respectively.

**Figure 1:**
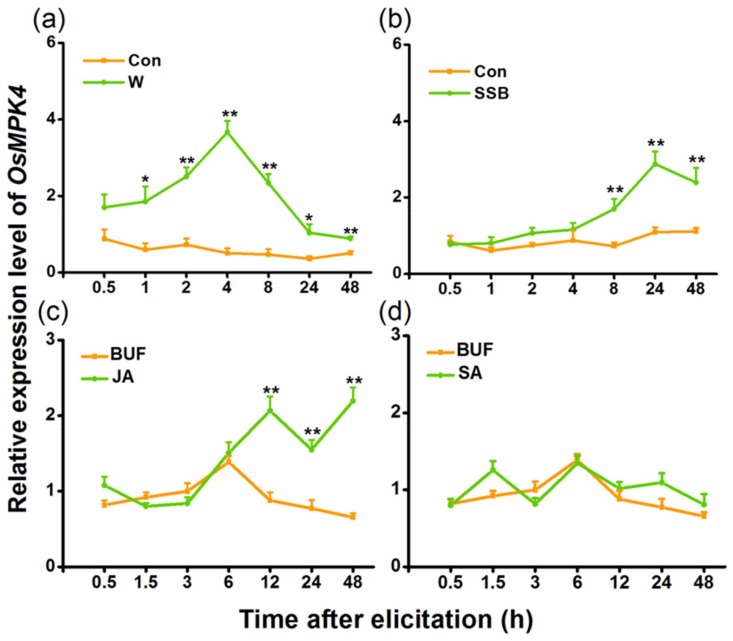
Relative transcript levels of *OsMPK4* in rice after different treatments. Mean transcript levels (+standard error (SE), *n* = 5) of *OsMPK4* in rice stems that were mechanically wounded (W) (**a**), infested by striped stem borer (SSB) (**b**), or treated with jasmonic acid (JA) (**c**) or salicylic acid (SA) (**d**). Transcript levels were analyzed by quantitative real-time PCR (qRT-PCR). Con, non-manipulated plant, BUF, buffer. Asterisks indicate significant differences in transcript levels between treatments and controls (*, *p* < 0.05; and **, *p* < 0.01, Student’s *t*-tests).

Quantitative real-time PCR (qRT-PCR) analysis revealed low constitutive levels of *OsMPK4* transcripts. Mechanical wounding, SSB infestation, and JA treatment markedly enhanced transcript levels of *OsMPK4*, whereas SA treatment did not (Figure 1). Moreover, unlike mechanical wounding, which quickly induced the expression of *OsMPK4*, SSB infestation and, especially, JA treatment increased the transcript level of *OsMPK4* only at later treatment stages. These data indicate that OsMPK4 might be involved in defense responses of rice to SSB.

### 2.2. Overexpression of OsMPK4

**Figure 2:**
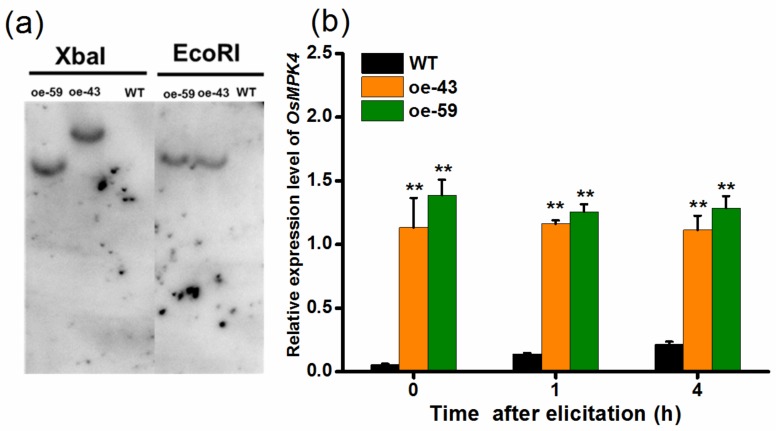
DNA gel-blot analysis of oe-MPK4 and expression levels of *OsMPK4* in transgenic lines and wild-type (WT) plants. (**a**) Genomic DNA was digested with *Eco*R I or *Xba* I. The blot was hybridized with a probe specific for the reporter gene encoding β-glucuronidase. Two transgenic lines (oe-43 and oe-59) have a single insertion of the transgene. The magnification is 1; (**b**) Mean transcript levels (+SE, *n* = 5) of *OsMPK4* in oe-MPK4 lines and WT plants that were individually wounded for 1 h and 4 h. Transcript levels were analyzed by qRT-PCR. Asterisks indicate significant differences in oe-MPK4 lines compared to WT plants (**, *p* < 0.01, Student’s *t*-tests).

**Figure 3:**
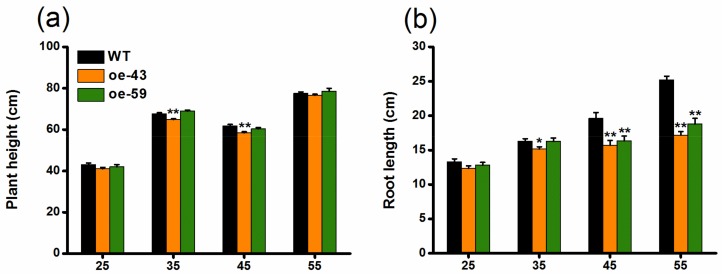

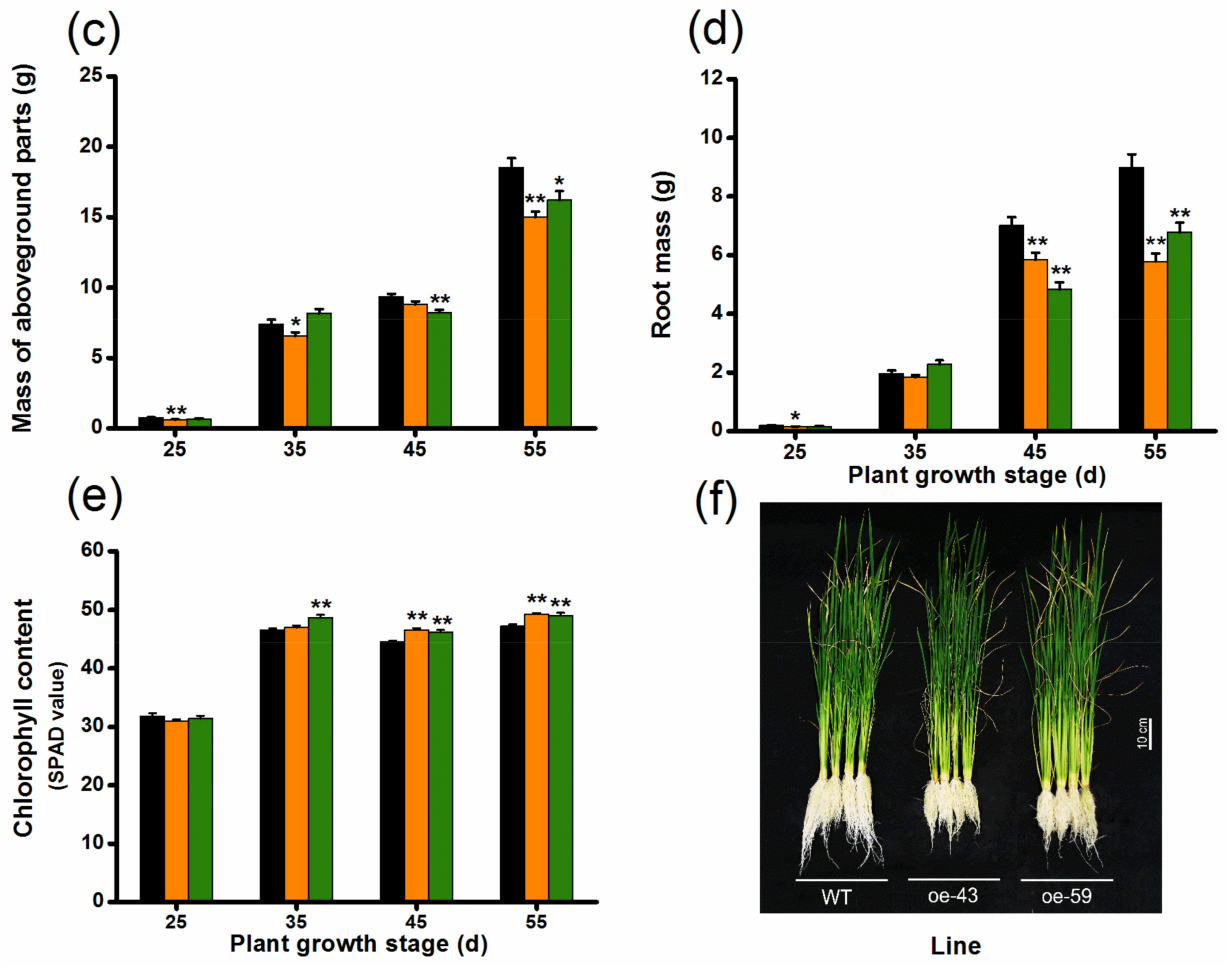
Growth phenotypes of oe-MPK4 lines and wild-type (WT) plants in the greenhouse. (**a**–**d**) Mean plant height (**a**) and root length (**b**) (+SE, *n* = 10) as well as mean mass (+SE, *n* = 10) of the aboveground part of plants (**c**) and roots (**d**) of oe-MPK4 lines and WT plants at 25, 35, 45, and 55 days; (**e**) Mean chlorophyll content (+SE, *n* = 30), measured by soil-plant analysis development (SPAD) meter, of oe-MPK4 lines and WT plants at 25, 35, 45, and 55 days; (**f**) Growth phenotype of 55 day old seedlings of oe-MPK4 lines and WT plants in the greenhouse. Asterisks indicate significant differences in oe-MPK4 lines compared to WT plants (*, *p* < 0.05; **, *p* < 0.01, Student’s *t*-tests).

To explore the function of OsMPK4 in herbivore-induced defenses, we obtained two homozygous single-insertion lines with over-expressed *OsMPK4* (oe-MPK4-43 and oe-MPK4-59) (Figure 2a). Transcript analysis found that constitutive transcript levels of *OsMPK4* in two oe-MPK4 lines, oe-MPK4-43 and oe-MPK4-59, were 20.4- and 25.0-fold higher, respectively, than levels of *OsMPK4* in wild type (WT) plants (Figure 2b); after mechanical wounding, transcript levels of *OsMPK4* in the two oe-MPK4 lines were still significantly higher than levels in WT plants: 4 h after mechanical wounding, for example, transcript levels of *OsMPK4* in the two oe-MPK4 lines were 5.2- and 6.0-fold higher than those in WT plants (Figure 2b). Lines with overexpressed *OsMPK4* showed growth retardation, especially at later growth stages (Figure 3a–d,f). At 55 days, for instance, the root length of the two oe-MPK4 lines, oe-MPK4-43 and oe-MPK4-59, decreased by approximately 31.85% and 25.35%, respectively, compared to that of WT plants (Figure 3b), and the total root mass of the two oe-MPK4 lines decreased by approximately 35.56% and 24.47% (Figure 3d); moreover, the mass of the aboveground part of oe-MPK4 plants decreased by approximately 18.96% and 12.46% (Figure 3c). Unlike the growth phenotype, the chlorophyll content in oe-MPK4 lines was a bit higher (about 4%) than in WT plants (Figure 3e). These results suggest that OsMPK4 is involved in rice growth and development.

### 2.3. Overexpressing OsMPK4 Increases Basal and/or Elicited Levels of JA, JA-Ile, Ethylene (ET), and Salicylic Acid (SA)

**Figure 4:**
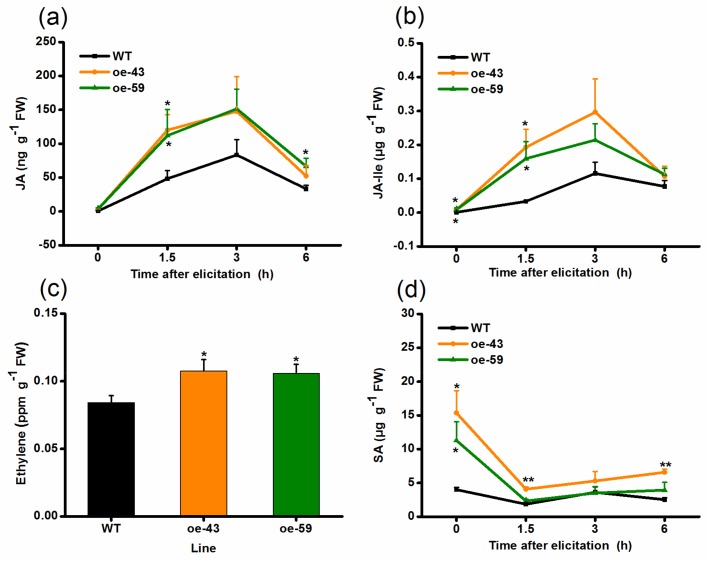
*OsMPK4* mediates SSB-induced JA, JA-Ile, ethylene, and SA accumulation. Mean levels (+SE, *n* = 5) of JA (**a**), JA-Ile (**b**), ethylene (**c**), and SA (**d**) in oe-MPK4 lines and wild-type (WT) plants that were individually infested by a third-instar SSB larva. Asterisks indicate significant differences in oe-MPK4 lines compared to WT plants (*, *p* < 0.05; **, *p* < 0.01, Student’s *t*-tests). FW, fresh weight.

Signaling pathways mediated by plant hormones, such as JA, ET, and SA, play key roles in herbivore-elicited defense responses in various plant species, including rice [6,34,35,36,37,38,43,44,45]. To evaluate whether *OsMPK4* affects the production of these plant hormones, and thus, regulates herbivore-induced defenses in rice, levels of these phytohormones were quantified in oe-MPK4 lines and WT plants. Basal JA and JA-Ile levels were similar between oe-MPK4 lines and WT plants, whereas levels of these two signals in oe-MPK4 lines were significantly increased compared to levels in WT plants after infestation by SSB: levels of JA and JA-Ile in the two oe-MPK4 lines were 2.48- and 2.32-fold, as well as 5.83- and 4.82-fold, respectively, higher than levels in WT plants at 1.5 h after SSB infestation (Figure 4a,b). Similarly, elicited levels of ET in oe-MPK4 lines were also higher than those in WT plants: 24 h after SSB infestation, ET accumulation in oe-MPK4 lines was increased by 25–28% compared to that in WT plants (Figure 4c). The overexpression of *OsMPK4* significantly enhanced constitutive levels of SA: levels in the two oe-MPK4 lines were 3.84- and 2.82-fold higher than levels in WT plants; after SSB infestation, the difference in the SA levels between oe-MPK4 lines and WT plants diminished, and only one oe-MPK4 line (oe-43) had higher elicited SA levels than did WT plants (Figure 4d). These results suggest that OsMPK4 modulates the biosynthesis of basal or SSB-elicited JA, JA-Ile, ET, and SA.

### 2.4. Overexpression of OsMPK4 Enhances SSB-Induced Levels of Trypsin Protease Inhibitors (TrypPIs) and Resistance to SSB

**Figure 5:**
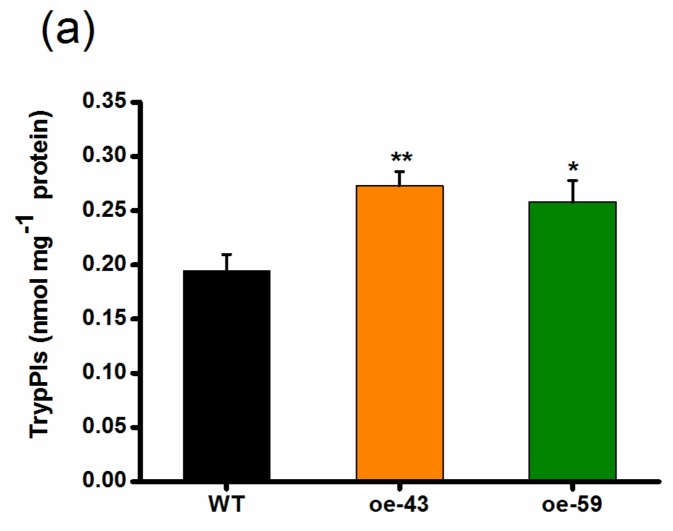

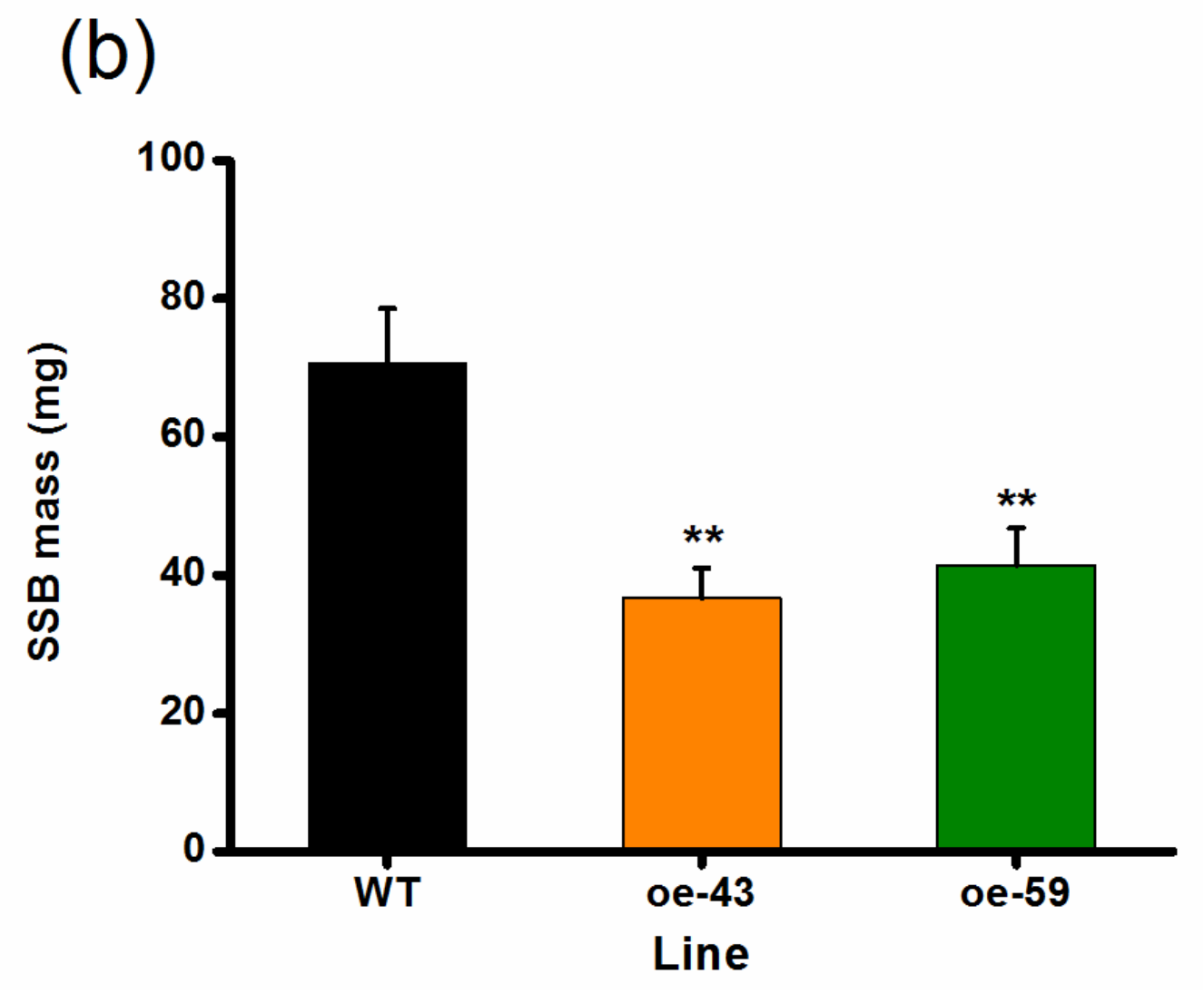
*OsMPK4* positively regulates SSB-induced trypsin protease inhibitor (TrypPI) accumulation and the resistance of rice to SSB. (**a**) Mean TrypPI activity (+SE, *n* = 5) in oe-MPK4 lines and wild-type (WT) plants that were individually infested by a third-instar SSB larva for 3 days; (**b**) Mean larval mass (+SE, *n* = 60) of SSB fed on oe-MPK4 lines and WT plants for 12 days. Asterisks indicate significant differences in oe-MPK4 lines compared to WT plants (*, *p* < 0.05; **, *p* < 0.01, Student’s *t*-tests).

TrypPIs, important direct defense compounds, help rice resist herbivores, especially chewing herbivores, and their production is positively regulated by both JA and ET signaling pathways [10,37,38]. Thus, we investigated if OsMPK4 modulates the production of TrypPIs and, eventually, the resistance of rice to the SSB. As expected, the activity of SSB-induced TrypPIs was higher in the two oe-MPK4 lines than that in WT plants (Figure 5a). Consistent with this, SSB caterpillars fed on oe-MPK4 lines gained significantly less mass compared with those fed on WT plants: the mass of larvae fed on the oe-MPK4 lines was only 52–59% of the mass of larvae fed on WT plants (Figure 5b). These data demonstrate that *OsMPK4* positively regulates SSB-induced TrypPIs and, thus, rice resistance to SSB.

## 3. Discussion

In the study, we found that *OsMPK4* could be induced by mechanical wounding, SSB infestation and JA treatment, but not by SA treatment (Figure 1). The overexpression of *OsMPK4* enhanced SSB-elicited and/or basal levels of JA, JA-Ile, ET, and SA in rice (Figure 4), which in turn, increased the activity of TrypPIs and the resistance of rice to the SSB (Figure 5a,b). In addition, the overexpression of *OsMPK4* impaired plant growth, shortening root lengths, and reducing stem and root biomasses (Figure 3b–d). The data demonstrate that *OsMPK4* functions as a negative regulator of plant growth but a positive regulator of SSB resistance in rice.

MPK4 has been reported to play an important but variable role in regulating defense-related signaling pathways in plants. In Arabidopsis, for instance, AtMPK4 negatively regulates SA levels, but positively modulates JA- and ET-mediated signaling pathways [28,29]. Similarly, NtMPK4 in *N. tabacum* inhibits the production of ozone-induced SA, but is required for the activation of the JA pathway [42]. By contrast, silencing *MPK4* in soybean enhances levels of pathogen-induced SA and simultaneously activates JA signaling [26]; in *N. attenuata*, NaMPK4 does not influence the biosynthesis of constitutive and herbivore-elicited SA, but does positively regulate the production of herbivore-induced JA [32]. Here, we found that SSB infestation induced the expression of *OsMPK4* (Figure 1b), and the overexpression of *OsMPK4* increased basal and/or herbivore-induced levels of SA, JA, JA-Ile, and ET in rice (Figure 4). This demonstrates that the function of OsMPK4 in rice defenses is different from its functions in other plant species. *OsMPK4* expression has previously been reported to be induced by *Xoo* infection and the overexpression of *OsMPK4* enhances constitutive and pathogen-elicited SA levels and JA levels in rice [31]. Moreover, JA treatment enhanced *OsMPK4* transcript levels only at late treatment stages, and SA treatment did not induce *OsMPK4* expression (Figure 1c,d). These findings indicate that OsMPK4 plays an important role in plant defenses against both pathogens and herbivores by functioning upstream of defense-related signaling pathways in a similar manner. That JA induces *OsMPK4* suggests that JA may also regulates the activity of this enzyme via positive feedback loops.

As stated above, many studies have demonstrated that MPKs can regulate levels of JA, SA, and ET in plants by influencing the activity of the enzymes related to signal biosynthesis [9,10,18,19]. Moreover, in Arabidopsis, AtMPK4 modulates SA-, JA-, and ET-dependent responses by suppressing the activity of enhanced disease susceptibility 1 (EDS1) and phytoalexin deficient 4 (PAD4), both of which act as activators of the SA pathway and repressors of the ET/JA pathway [29]. Thus, in rice, the regulation of OsMPK4 on these defense-related signaling pathways may occur via similar mechanisms. Interestingly, OsMPK3 has also been found to mediate the biosynthesis of herbivore-induced JA and the resistance of rice to herbivores [10]. This indicates that there is an overlap in the function of the two MPKs in the herbivore-induced defense responses in rice. Future research should elucidate how the two MPKs regulate rice defenses, and what the similarities and the differences are between them.

Accumulated evidence demonstrates that there is antagonism between the JA and SA pathways in many plant species, including rice [38,46,47]. However, we found that the overexpression of *OsMPK4* enhanced basal and/or herbivore-elicited levels of both SA and JA (Figure 4); this finding was consistent with the previously reported result that rice lines that overexpressed *OsMPK4* had higher basal and/or pathogen-induced levels of SA and JA than did WT plants [31]. A similar result was observed in soybean [26]. Complicating the results, knocking out or silencing *OsMPK4* enhances constitutive and pathogen-elicited SA levels, but slightly decreases pathogen-elicited JA levels, suggesting there is antagonism between the SA and JA pathways [31,48]. The complexity of the findings indicates that, in addition to acting as a regulator of SA–JA antagonism, MPK4 may also function as a regulator of SA and JA synergism in some plant species or under some conditions. Further research should elucidate these mechanisms.

JA and ET signaling pathways have been reported to positively mediate the production of TrypPIs, a kind of direct defense compound that protects rice plants against chewing herbivores, including the SSB [37,38,49]. Therefore, enhanced resistance of oe-MPK4 lines to SSB is probably because of higher levels of JA, JA-Ile, and ET in these lines compared to WT plants; these signals led to increased activity among defensive compounds, including TrypPIs in oe-MPK4 lines.

In addition to its role in plant defense responses, OsMPK4 plays a role in plant growth. In Arabidopsis and soybean (*G. max*), for instance, the *mpk4* mutant is severely dwarfed [26,27]. In the wild tobacco species *N. attenuata*, silencing *NaMPK4* causes plants to be somewhat small in stature [50]. Consistent with these data, the overexpression of *BnMPK4* in *B. napus* increases plant size [41]. Research into the mechanisms underlying the growth phenotype of *mpk4* mutants revealed that elevated constitutive defense responses [28], ROS homeostasis deregulation, and photosynthesis damage [27], auxin repression [26], or rapid water loss [50] may explain why these mutants are so small. Unlike the positive effect of MPK4 on plant growth found in other species, we found that the overexpression of *OsMPK4* retarded rice growth moderately (Figure 3). This result may be related to the function of OsMPK4 in rice, which is different from that of MPK4 in other plant species, as stated above: the overexpression of *OsMPK4* enhanced constitutive SA levels (Figure 4d). Given that the growth retardation of the *mpk4* mutant in Arabidopsis is partly related to the accumulation of SA [28], the possibility could not be ruled out that the reduced size of mutants might be, at least in part, due to their higher SA levels than WT plants.

In summary, our findings demonstrate that OsMPK4 plays an important role in plant growth and herbivore-induced defense responses of rice by regulating SA-, JA-, and ET-mediated signaling pathways, and that the function of OsMPK4 in rice, a positive regulator of plant herbivore resistance and a negative regulator of plant growth, is different from the function of MPK4 reported in other plant species, such as Arabidopsis and soybean. Moreover, OsMPK4 may also mediate SA–JA/ET interactions in rice, depending on the stimulus: a stimulus that suppresses the activity of OsMPK4 will cause SA–JA/ET antagonism, whereas a stimulus that induces the activity of OsMPK4 will result in SA–JA/ET synergism; in other words, OsMPK4 may also play an important role in modulating appropriate and specific defense responses in plants to different stresses.

## 4. Materials and Methods

### 4.1. Plant Growth

The rice (*Oryza sativa*) genotypes used in this study were cv Xiushui 11 wild-type (WT) and two oe-MPK4 transgenic lines (see below). Pre-germinated seeds of WT plants and transgenic lines were cultured in plastic cups (diameter, 8 cm; height, 10 cm) in a climate incubator at 28 ± 2 °C, with a 14 h light phase. Twelve day old seedlings were transferred to 25 L hydroponic boxes with a rice nutrient solution [51]. After 30–35 days, plants were transplanted in individual 500 mL hydroponic plastic pots. Plants were used for experiments 4–5 days after transplanting.

### 4.2. Insects

An SSB colony was originally obtained from a rice field in Hangzhou, China, and maintained on TN1 (a rice variety that is susceptible to SSB) rice seedlings in a controlled climate chamber at 26 ± 2 °C, with a 14 h light phase and 65% relative humidity.

### 4.3. Isolation and Characterization of OsMPK4 cDNA

The full-length cDNA of *OsMPK4* was PCR-amplified. The primers MPK4-F (5′-ATAGTCGACTCCACCTCGTCCT-3′) and MPK4-R (5′-ATATCTAGAAGGGGATTTGGCTTT-3′) were designed based on the sequence of *OsMPK4*. The PCR products were cloned into the pMD19-T vector (TaKaRa, Tokyo, Japan) and sequenced.

### 4.4. Phylogenetic Analysis

For the phylogenetic analysis, the program MEGA 6.0 [52] was used. Protein sequences were downloaded from GenBank (https://www.ncbi.nlm.nih.gov/genbank/) and were aligned using the ClustalW algorithm in MEGA 6.0 (Tempe, AZ, USA). This alignment was then used to generate an unrooted tree using the maximum-likelihood approach [53] (1000 replications).

### 4.5. Generation and Characterization of Transgenic Plants

The full-length cDNA sequence of *OsMPK4* was cloned into the pCAMBIA1301 transformation vector to yield an overexpressed construct containing the hygromycin resistance gene *hph* and the reporter gene encoding β-glucuronidase (GUS) (GenBank: AF234297) as selectable markers (Figure S3). The vector was used for transforming the rice variety Xiushui 11 by *Agrobacterium*-mediated transformation system. Homozygous T2 transgenic plants were selected using GUS staining or hygromycin resistance screening [38]. Two T2 homozygous lines (oe-43 and oe-59), each harboring a single insertion (Figure 2a), were used in subsequent experiments.

### 4.6. Plant Treatments

For mechanical wounding, plants were individually pierced with a needle on the lower part of leaf sheaths (about 2 cm long), each 200 times (W). Control plants were not manipulated (C). For SSB treatment, plants were individually infested with a third-instar SSB larva that had been starved for 2 h. Non-infested plants were used as controls (C). For JA and SA treatment, each plant was sprayed with 2 mL of JA (100 μg·mL^−1^) or SA (70 μg·mL^−1^) in 50 mM sodium phosphate buffer (pH 8, with 0.01% Tween). Plants sprayed with 2 mL of the buffer (BUF) were used as controls. Each treatment at each time point was replicated five times.

### 4.7. Measurement of Plant Growth Parameters

Plant growth parameters, including plant height, root length, mass of the above- and belowground part of a plant, and chlorophyll content, of oe-WPK4 lines and WT plants, were measured at 25, 35, 45, and 55 days after plant growth. Plant height and root length were defined as the part of a plant from the stem base to the longest leaf apex and that from the stem base to the longest root tip, respectively. Plants were cut off from the stem base and then the mass of aboveground and belowground (roots) part of plants was measured. For chlorophyll content determination, three leaves with identical leaf positions from each plant and three identical locations from each leaf were measured by a soil-plant analysis development (SPAD) meter. Every experiment at each stage was replicated ten times.

### 4.8. JA, JA-Ile, and SA Analysis

For JA, JA-Ile, and SA analysis, plants of the different genotypes were randomly assigned to SSB and control treatments. Plant stems were harvested at different time points after the start of treatment (see details in Figure 4). Samples (each with about 150 mg) were ground in liquid nitrogen, and JA, JA-Ile, and SA were extracted with ethyl acetate spiked with labeled internal standards (D6-JA, D6-JA-Ile, D4-SA) and analyzed with an HPLC/mass spectrometry/mass spectrometry system following the method described in Lu et al. [54]. Each treatment at each time point was replicated five times.

### 4.9. Ethylene Analysis

Plants of WT and transgenic lines were randomly assigned to SSB and control treatments, and were individually confined with sealed glass cylinders (diameter 4 cm, height 50 cm). The production of ethylene was determined following the same method as described in Lu et al. [55]. Each treatment at each time point was replicated five times.

### 4.10. TrypPI Activity Analysis

Plants of different genotypes were randomly assigned to SSB and control treatments. Stems of oe-MPK4 lines and WT plants (about 0.3 g per sample) were individually harvested 3 days after the start of the treatment. TrypPI concentrations were measured using a radial diffusion assay as described by van Dam et al. [56]. Each treatment was replicated five times.

### 4.11. SSB Performance Measurement

The performance of SSB larvae on oe-MPK4 lines and WT plants was determined by releasing one freshly hatched larva onto each plant. Larval mass (to an accuracy of 0.1 mg) was measured and recorded 12 days after the release of the herbivore. Each treatment was replicated thirty times.

### 4.12. Data Analysis

Differences in data in different lines or treatments were determined by analyzing variance followed by Duncan’s multiple range test (or Student’s *t*-test for comparing two treatments). When necessary, data were log-transformed or arcsine-transformed to meet requirements for the homogeneity of variance. All tests were carried out with SPSS software version 20.

## Supplementary Materials

Supplementary materials can be found at http://www.mdpi.com/1422-0067/19/4/1182/s1.

## Abbreviations

MPKs	mitogen-activated protein kinases
JA	jasmonic acid
JA-Ile	jasmonoyl-l-isoleucine
SA	salicylic acid
ET	ethylene
TrypPIs	trypsin proteinase inhibitors
SSB	striped stem borer
