# Supplementary material for: Expressing OsMPK4 Impairs Plant Growth but Enhances the Resistance of Rice to the Striped Stem Borer Chilo suppressalis
Source: Int J Mol Sci. 2018 Apr 13;19(4):1182. doi: 10.3390/ijms19041182 (PMC5979284; doi:10.3390/ijms19041182)
Supplement: Supplementary file 1 [file ijms-19-01182-s001.pdf]

Supplementary Materials: Expressing *OsMPK4* Impairs Plant Growth but Enhances the Resistance of Rice to the Striped Stem Borer *Chilo suppressalis*

Xiaoli Liu, Jiancai Li, Liping Xu, Qi Wang and Yonggen Lou*

```
   1 ATGGATTCCTCCTCCGGCGGCGCGGGCGGCGGCGGCGGCGCGCAGATCAAGGGGATGGGG
   1  M  D  S  S  S  G  G  A  G  G  G  G  G  A  Q  I  K  G  M  G
  61 ACGCACGGGGGCCGCTACGTGCTGTACAACGTGTACGGGAACTTCTTCGAGGTCTCCTCC
  21  T  H  G  G  R  Y  V  L  Y  N  V  Y  G  N  F  F  E  V  S  S
 121 AAGTACGCCCCTCCCATCCGCCCCATCGGCCGGGGCGCCTACGGCATTGTCTGCGCGGCT
  41  K  Y  A  P  P  I  R  P  I  G  R  G  A  Y  G  I  V  C  A  A
 181 GTTAACTCGGAGAACGGCGAGGAAGTTGCCATCAAGAAGATTGGCAATGCATTCGACAAC
  61  V  N  S  E  N  G  E  E  V  A  I  K  K  I  G  N  A  F  D  N
 241 CATATCGATGCCAAGCGGACACTGAGAGAAATCAAGCTGCTTCGCCACATGGACCACGAG
  81  H  I  D  A  K  R  T  L  R  E  I  K  L  L  R  H  M  D  H  E
 301 AATATTATTGCCATAAAGGACATAATTCGCCCCCCAAGAAGAGACAACTTTAATGATGTT
 101  N  I  I  A  I  K  D  I  I  R  P  P  R  R  D  N  F  N  D  V
 361 TACATTGTTTCTGAGTTGATGGATACTGATCTCCATCAGATCATACGCTCAAATCAACCA
 121  Y  I  V  S  E  L  M  D  T  D  L  H  Q  I  I  R  S  N  Q  P
 421 TTGACTGATGACCACTGCCAGTACTTCCTGTACCAGTTGCTACGAGGGCTAAAATATGTG
 141  L  T  D  D  H  C  Q  Y  F  L  Y  Q  L  L  R  G  L  K  Y  V
 481 CACTCGGCAAATGTCTTGCACCGTGATCTGAAGCCAAGCAATTTGTTCCTTAATGCAAAT
 161  H  S  A  N  V  L  H  R  D  L  K  P  S  N  L  F  L  N  A  N
 541 TGTGATCTCAAGATTGCTGATTTTGGGCTTGCAAGAACCACTACGGAGACTGACCTCATG
 181  C  D  L  K  I  A  D  F  G  L  A  R  T  T  T  E  T  D  L  M
 601 ACAGAGTATGTGGTCACTCGTTGGTATCGAGCACCAGAGCTGCTGTTGAACTGCTCGCAG
 201  T  E  Y  V  V  T  R  W  Y  R  A  P  E  L  L  L  N  C  S  Q
 661 TATACTGCTGCTATTGATGTCTGGTCAGTTGGATGCATACTTGGTGAAATTGTGACTCGT
 221  Y  T  A  A  I  D  V  W  S  V  G  C  I  L  G  E  I  V  T  R
 721 CAACCCCTGTTTCCTGGAAGGGATTACATTCAGCAACTAAAATTGATCACTGAGCTGATA
 241  Q  P  L  F  P  G  R  D  Y  I  Q  Q  L  K  L  I  T  E  L  I
 781 GGGTCGCCAGATGACTCAAGCCTAGGGTTTCTTCGGAGTGATAATGCAAGAAGATACATG
 261  G  S  P  D  D  S  S  L  G  F  L  R  S  D  N  A  R  R  Y  M
 841 AAACAGCTACCACAGTACCCAAGGCAGGACTTCCGCTTGCGCTTCCGCAACATGTCTGCT
 281  K  Q  L  P  Q  Y  P  R  Q  D  F  R  L  R  F  R  N  M  S  A
 901 GGTGCAGTCGATCTGTTAGAGAAAATGCTGGTGTTTGACCCAAGCAGACGGATAACTGTT
 301  G  A  V  D  L  L  E  K  M  L  V  F  D  P  S  R  R  I  T  V
 961 GATGAGGCTCTTCATCACCCATACTTGGCTTCTCTTCATGACATCAATGAAGAACCCACC
 321  D  E  A  L  H  H  P  Y  L  A  S  L  H  D  I  N  E  E  P  T
1021 TGCCCAGCACCTTTCAGCTTTGATTTTGAGCAACCATCCTTTACTGAAGAACATATAAAA
 341  C  P  A  P  F  S  F  D  F  E  Q  P  S  F  T  E  E  H  I  K
1081 GAACTCATCTGGAGGGAATCCTTGGCATTTAATCCGGATCCTCCCTACTAA
 361  E  L  I  W  R  E  S  L  A  F  N  P  D  P  P  Y  *
```

Figure S1. Sequences of nucleotides and deduced amino acids of *OsMPK4*. The stop codon is marked by an asterisk.

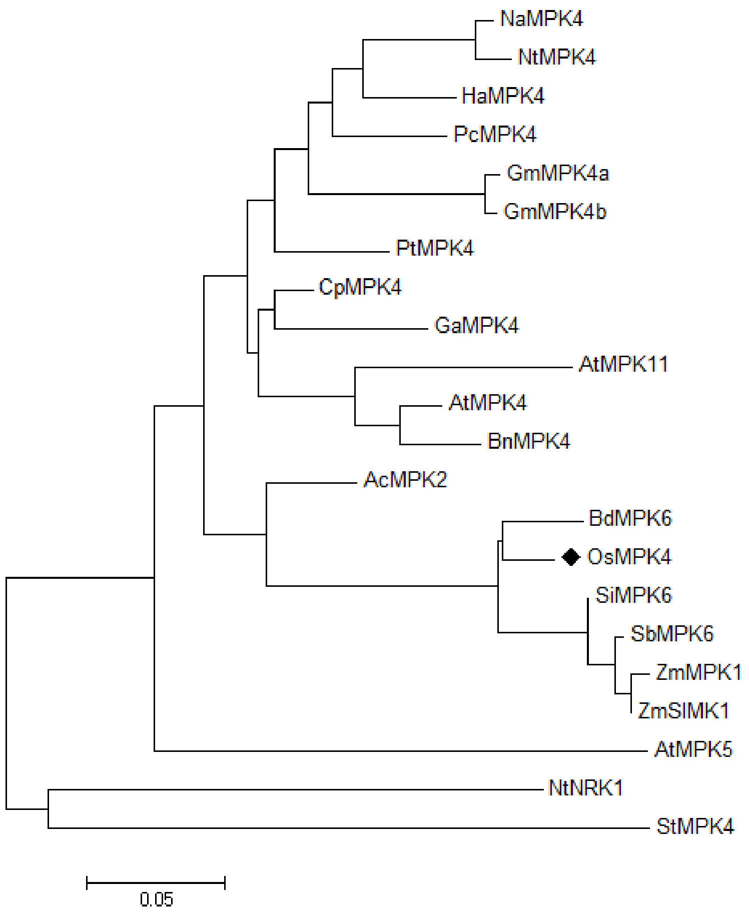

Figure S2. Phylogenetic analysis of group B-type MAPKs from different species. The unrooted tree was constructed with a maximum likelihood method on the basis of the alignment of protein sequences and confirmation of the tree topology by bootstrap analysis (1000 replicates) were performed with MEGA software (default settings except the replicates of the bootstrap value). Species acronyms are included before the protein name: Ac, *Ananas comosus*; At, *Arabidopsis thaliana*; Bd, *Brachypodium distachyon*; Bn, *Brassica napus*; Cp, *Carica papaya*; Ga, *Gossypium arboreum*; Gm, *Glycine max*; Ha, *Helianthus annuus*; Na, *Nicotiana attenuata*; Nt, *Nicotiana tabacum*; Os, *Oryza sativa*; Pc, *Petroselinum crispum*; Pt, *Populus trichocarpa*; Sb, *Sorghum bicolor*; Si, *Setaria italica*; St, *Solanum tuberosum*; Zm, *Zea mays*. The scale bar represents 0.05 amino acid substitutions per site in the primary structure. Sequence data in the phylogenic tree can be found in the GenBank/EMBL data libraries under accession numbers: AcMPK2 (XP_020080580.1); AtMPK4 (NP_192046.1); AtMPK5 (NP_567378.4); AtMPK11 (NP_001117210.1); BdMPK6 (XP_003574247.1); BnMPK4 (NP_001303223.1); CpMPK4 (XP_021887822.1); GaMPK4 (KHG15311.1); GmMPK4a (NP_001339892.1); GmMPK4b (NP_001242364.2); HaMPK4 (XP_021969289.1); NaMPK4 (ABO65100.1); NtMPK4 (NP_001312502.1); NtNRK1 (BAB32406.1); OsMPK4 (XP_015615011.1); PcMPK4 (AAN65180.1); PtMPK4 (XP_002302599.1); SbMPK6 (XP_002467591.1); SiMPK6 (XP_004983829.1); StMPK4 (BAB93529.1); ZmMPK1 (AIX47138.1); ZmMPK4 (BAA74733.1); ZmSIMK1 (NP_001105239.2). The diamond indicates the gene OsMPK4 that is characterized in this paper.

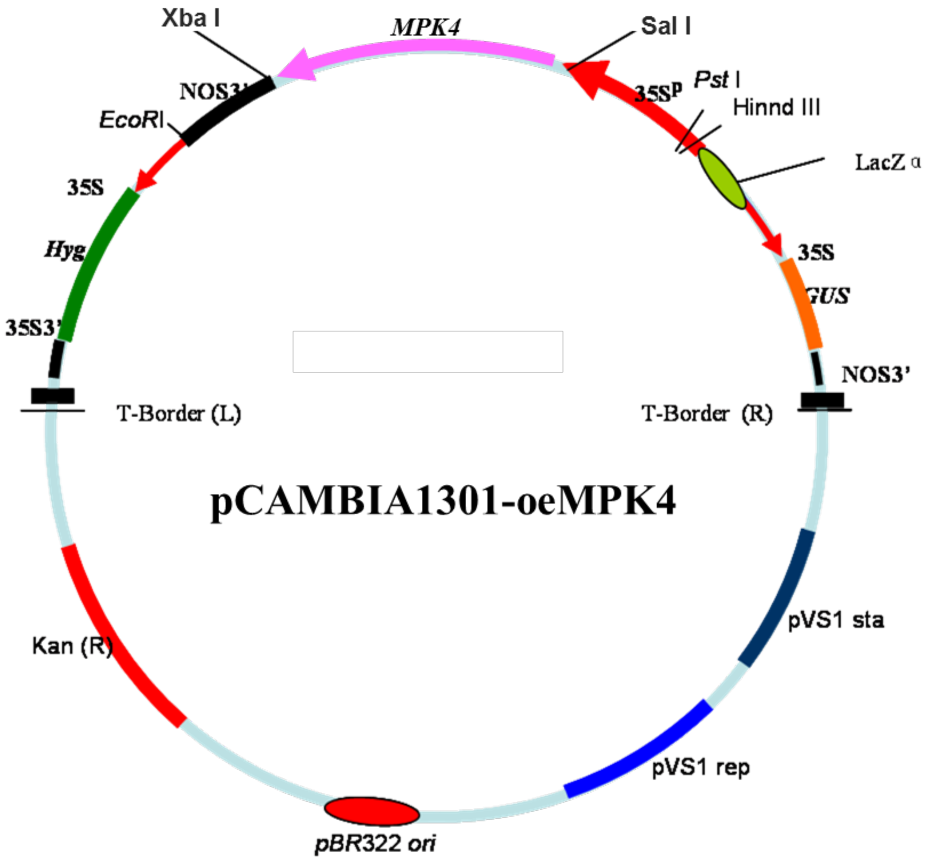

Figure S3. Transformation vector used in this study
